# Gain of Function of Ion Channel TRPV1 Exacerbates Experimental Colitis by Promoting Dendritic Cell Activation

**DOI:** 10.1016/j.omtn.2020.10.006

**Published:** 2020-10-14

**Authors:** Lina Duo, Ting Wu, Ziliang Ke, Linghan Hu, Chaohui Wang, Guigen Teng, Wei Zhang, Weihong Wang, Qing Ge, Yong Yang, Yun Dai

**Affiliations:** 1 Department of Dermatology, Peking University First Hospital, Beijing Key Laboratory of Molecular Diagnosis on Dermatoses, Beijing, China; 2 Department of Gastroenterology, Peking University First Hospital, Beijing, China; 3 Department of Dermatology, Chengdu Integrated TCM & Western Medicine Hospital, Chengdu, China; 4 Peking-Tsinghua Center for Life Sciences, Beijing, China; 5 Academy for Advanced Interdisciplinary Studies, Peking University, Beijing, China; 6 Department of Immunology, Peking University Health Science Center, Beijing, China; 7 Institute of Dermatology, Chinese Academy of Medical Sciences and Peking Union Medical College, Nanjing, Jiangsu, China

**Keywords:** TRPV1, gain of function, colitis, dendritic cells, Th17 cells, NFATc2

## Abstract

Dysregulated mucosal immunity plays an essential role in the pathophysiology of inflammatory bowel disease (IBD). Transient receptor potential vanilloid 1 (TRPV1) is a Ca^2+^-permeable ion channel that is implicated in modulating immune responses. However, its role in the pathogenesis of intestinal inflammation remains elusive. Here, we found that TRPV1 gain of function significantly increased the susceptibility of mice to experimental colitis, and that was associated with excessive recruitment of dendritic cells and enhanced Th17 immune responses in the lamina propria of colon. TRPV1 gain of function promoted dendritic cell activation and cytokine production upon inflammatory stimuli, and consequently enhanced dendritic cell-mediated Th17 cell differentiation. Further mechanistic studies showed that TRPV1 gain of function in dendritic cells enhanced activation of calcineurin/nuclear factor of activated T cells (NFATc2) signaling induced by inflammatory stimuli. Moreover, in patients with IBD, TRPV1 expression was increased in lamina propria cells of inflamed colon compared with healthy controls. Our findings identify an important role for TRPV1 in modulating dendritic cell activation and sustaining Th17 responses to inflammatory stimuli, which suggest that TRPV1 might be a potential therapeutic target in controlling mucosal immunity and IBD.

## Graphical Abstract

**Figure:**
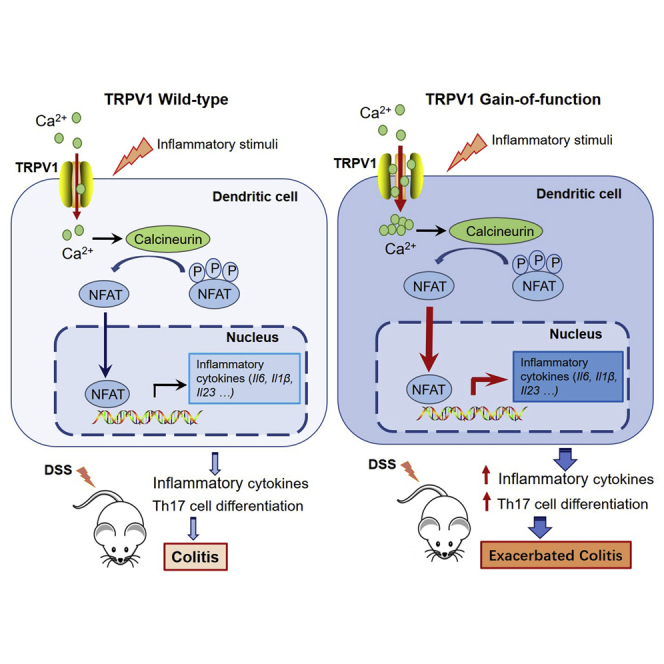

## Teaser

Transient receptor potential vanilloid 1 (TRPV1) is a Ca^2+^-permeable ion channel mostly known as a nociceptive receptor in neurons. By using a novel murine model, the authors showed that TRPV1 gain of function enhances dendritic cell activation upon inflammatory stimuli, and consequently sustains Th17 cell differentiation and colitogenic responses.

## Introduction

Inflammatory bowel disease (IBD) is a chronic inflammatory condition of the gastrointestinal tract, mainly including Crohn’s disease (CD) and ulcerative colitis (UC). Although the etiology of IBD is currently unclear, this disease is generally thought to develop from dysregulated immune responses toward gut microbiota or their products.^1^ The pathological immune responses are characterized by a pronounced infiltration of antigen-presenting cells (APCs) and T cells in the inflamed sites of IBD patients. Among infiltrating APCs, dendritic cells (DCs) are essential in the initiation and regulation of mucosal immunity.^2^ Following pathogen recognition through pattern recognition receptors (PRRs), DCs upregulate costimulatory molecules and major histocompatibility complex molecule class II (MHC II) and produce cytokines that control differentiation of naive CD4^+^ T cells into effector lineages. The pathogenic role of DCs in IBD development has been well established.^1^^,^^3^

Transient receptor potential vanilloid 1 (TRPV1) is a member of the TRP family of channels, which is a nonselective cation channel with high Ca^2+^ permeability and was initially identified as the receptor for capsaicin.^4^ TRPV1 can be activated by a variety of physical and chemical stimuli that are responsible for nociceptive, thermal, and mechanical sensations.^4^^,^^5^ Once believed to be exclusively associated with sensory neurons, TRPV1 expression is now demonstrated in non-neuronal cells of almost all organs.^6^ TRPV1 has been implicated in the pain sensation in various gastrointestinal disorders, including gastroesophageal reflux disease and irritable bowel syndrome.^7^^,^^8^ In IBD patients, TRPV1-positive nerve fibers are substantially increased in colon, which is correlated with abdominal pain severity.^9^^,^^10^ Although accumulating evidence indicates that TRPV1 can be activated and sensitized in inflammatory conditions,^11^^,^^12^ the expression of TRPV1 protein in non-neuronal cells of human inflamed intestine and its role in the pathogenesis of IBD remain unclear.

TRPV1 is expressed in almost all types of mammalian immune cells, including DCs, macrophages, lymphocytes, and neutrophils.^13^^,^^14^ TRPV1 channel activation triggers influx of extracellular Ca^2+^ into cells, and the increase in intracellular Ca^2+^ activates protein kinases, including Ca^2+^/calmodulin-dependent protein kinases and mitogen-activated protein kinase (MAPK), particularly extracellular signal-regulated kinase (ERK) and p38 MAPK.^15^ In addition, stimulation of the TRPV1 channel activates stimulus-responsive transcription factors, such as activator protein 1 (AP-1), cAMP-response element-binding protein (CREB), and nuclear factor of activated T cells (NFATs), and these in turn lead to activation, proliferation, and differentiation of immune cells.^13^^,^^16^ Previous study has revealed that TRPV1 is functionally expressed in CD4^+^ T cells and promotes the activation and pro-inflammatory properties of CD4^+^ T cells.^14^ The wide distribution of TRPV1 in immune cells suggests its role in modulating inflammatory responses. However, the reported role of TRPV1 in colitis development is sometimes contradictory. Both the pro- and anti-inflammatory effects of TRPV1 have been described in a TRPV1 deficiency mouse model for colitis.^17^, ^18^, ^19^ Moreover, the TRPV1-independent effects of currently available TRPV1 agonists and antagonists may give a misleading result in some studies.^20^^,^^21^ In terms of mechanism, the consequences of a channel-sustained opening may be far more significant than changes in its expression. Thus, we generated TRPV1 gain-of-function mutation mice, which carry a homozygous glycine-to-serine substitution at amino acid 564 (*Trpv1*^G564S+/+^) to render the channel constitutively active.^22^ Here, we used it as a tool to elucidate the role of endogenous TRPV1 channel activity in the pathogenesis of colon inflammation. To verify whether our findings in mice have relevance to human IBD, we determined the protein level of TRPV1 in colon tissues from IBD patients and healthy controls.

## Results

### TRPV1 Gain of Function Exacerbates Dextran Sulfate Sodium (DSS)-Induced Colitis

**Figure 1:**
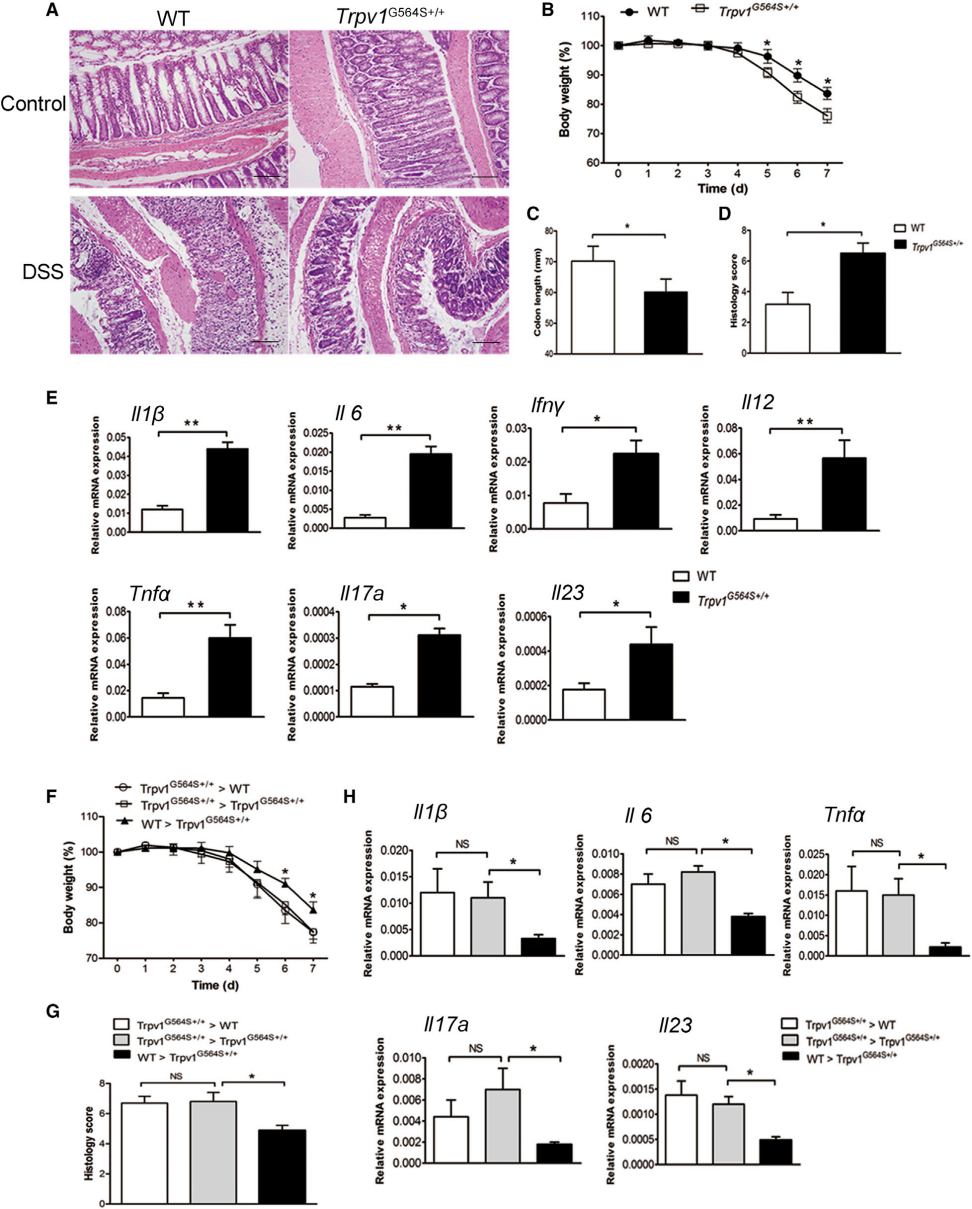
TRPV1 Gain of Function Exacerbates Experimental Colitis, and Hematopoietic Cells Are Essential to Confer Enhanced Susceptibility to Inflammatory Stimuli (A–E) Wild-type (WT) and *Trpv1*^G564S+/+^ mice were treated with or without 2% DSS for 7 days. (A) Representative hematoxylin and eosin-stained sections of colons from the indicated groups. (B) Mean change in body weight (relative to starting weight, set as 100%) throughout the study. (C–E) Colon length (C), microscopic inflammation score (D), and relative mRNA expression of *Il1β*, *Il6*, *Ifnγ*, *Il12*, *Tnfα*, *Il17a*, and *Il23* in colon tissues (E) of indicated groups on day 7 of DSS treatment. (F–H) Mice were lethally irradiated and reconstituted for 8 weeks with *Trpv1*^G564S+/+^ or WT bone marrow cells and treated with 2% DSS for 7 days. Mean change in body weight (F), microscopic inflammation score (G), and relative mRNA expression of *Il1β*, *Il6*, *Tnfα*, *Il17a*, and *Il23* in colon tissues (H) of indicated groups. All data are represented as mean ± SEM; n = 8–12 per group. ∗p < 0.05, ∗∗p < 0.01. Scale bar, 100 μm. NS, not significant.

*Trpv1*^G564S+/+^ mice developed normally and displayed no obvious pathological changes in major organs, including liver, kidney, lung, and spleen. Moreover, *Trpv1*^G564S+/+^ mice had normal colonic crypt morphology and did not develop spontaneous colitis (Figure 1A). DSS causes chemical injury to colonic mucosa and results in the exposure of the lamina propria and submucosa to luminal antigens and bacteria, which leads to colon inflammation. After treatment with 2% DSS for 7 days, *Trpv1*^G564S+/+^ mice displayed more rapid body weight loss (Figure 1B) and significant inflammation-induced colonic shorting as compared with their wild-type (WT) littermates (Figure 1C). Histological analysis showed a more severe form of acute colitis in *Trpv1*^G564S+/+^ mice, with extensive epithelial denudation and prominent inflammatory cell infiltration (Figure 1A). There was an elevated microscopic inflammatory score in *Trpv1*^G564S+/+^ mice compared with the WT group (Figure 1D). Moreover, the expression of pro-inflammatory cytokines *Il6*, *Il1β*, *Il12*, *tumor necrosis factor α (Tnfα)*, *interferon γ (Ifnγ)*, *I17a*, and *Il23* in colon tissues from *Trpv1*^G564S+/+^ mice was higher than that from WT littermates (Figure 1E). These results suggested that TRPV1 gain of function increases the susceptibility of mice to DSS-induced colitis.

### Hematopoietic TRPV1 Gain of Function Is Essential to Confer Enhanced Susceptibility to Colitis

Because *Trpv1*^G564S+/+^ mice show mutation of TRPV1 in all cell types, we questioned which cell fraction may render mice more susceptible to colitis. *Trpv1*^G564S+/+^ and WT mice were irradiated and intravenously injected with *Trpv1*^G564S+/+^ bone marrow (BM) cells to generate *Trpv1*^G564S+/+^ > *Trpv1*^G564S+/+^ and *Trpv1*^G564S+/+^ > WT BM chimeras. After 2% DSS treatment for 7 days, *Trpv1*^G564S+/+^ > WT chimeras had colitis development similar to that of *Trpv1*^G564S+/+^ > *Trpv1*^G564S+/+^ chimeras in terms of body weight loss (Figure 1F) and histological inflammation score (Figure 1G), suggesting that colonic epithelial cells were not the primary targets for TRPV1 gain of function to confer enhanced susceptibility to colitis. We also reconstituted irradiated *Trpv1*^G564S+/+^ mice with BM cells from WT or *Trpv1*^G564S+/+^ mice. Notably, WT > *Trpv1*^G564S+/+^ chimeras were significantly protected from DSS-induced colitis, which was shown by reduced weight loss (Figure 1F) and inflammation score as compared with *Trpv1*^G564S+/+^ > *Trpv1*^G564S+/+^ chimeras (Figure 1G). The expressions of *Il6*, *Il1β*, *Tnfα*, *Il17a*, and *Il23* in colon tissues from WT > *Trpv1*^G564S+/+^ chimeras were also remarkably reduced as compared with *Trpv1*^G564S+/+^ > *Trpv1*^G564S+/+^ chimeras (Figure 1H). These findings conclusively demonstrated that TRPV1 gain of function in hematopoietic cells, but not in epithelial cells, is responsible for the increased susceptibility to colitis.

### TRPV1 Gain of Function Promotes Recruitment and Activation of DCs in Colitis

**Figure 2:**
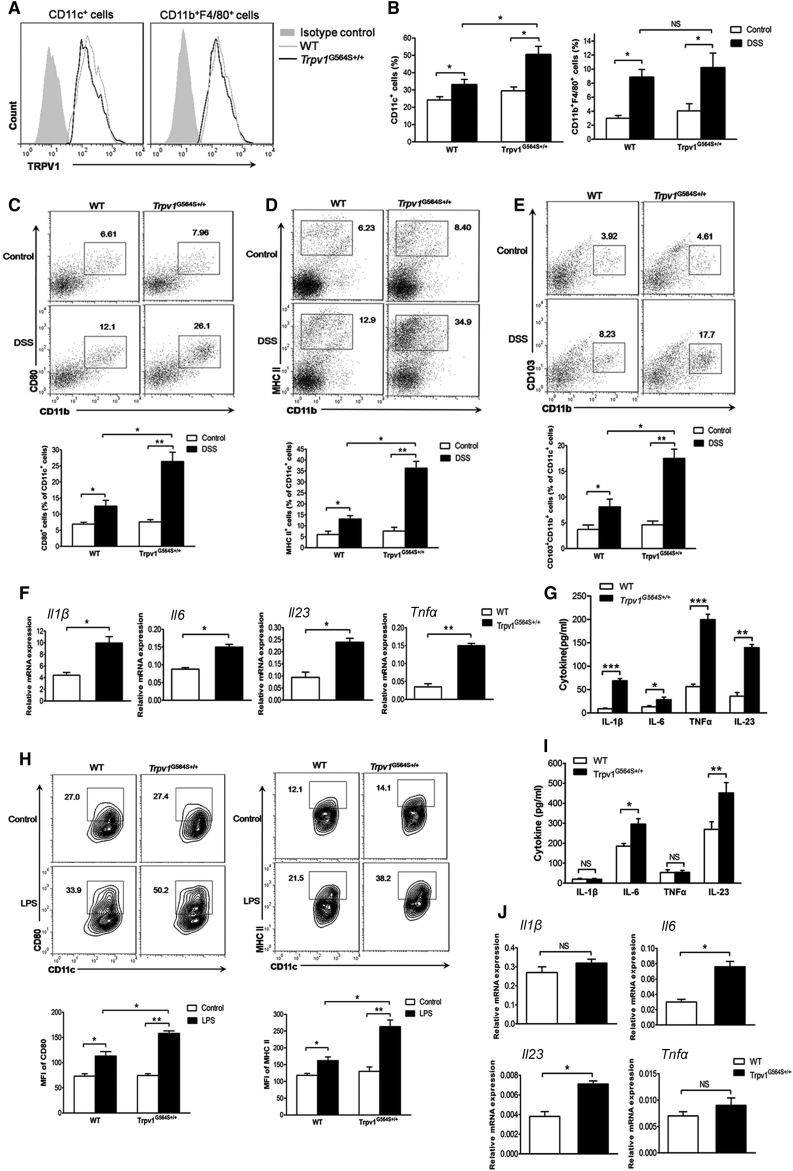
TRPV1 Gain of Function Promotes Accumulation and Activation of DCs (A) Flow cytometry analysis of TRPV1 expression in DCs (CD11c^+^) and macrophages (CD11b^+^F4/80^+^) in colonic lamina propria of WT and *Trpv1*^G564S+/+^ mice. (B–E) Colonic lamina propria mononuclear cells were isolated from WT and *Trpv1*^G564S+/+^ mice treated with or without 2% DSS for 7 days. (B) Quantification of DCs and macrophages in total viable cells by flow cytometry. (C–E) Representative flow cytometry plots (top; gated on CD11c^+^ cells) and quantification (bottom) of CD11c^+^CD80^+^ (C), CD11c^+^MHCII^+^ (D), and CD11c^+^CD11b^+^CD103^+^ (E) DC subsets. (F and G) DCs were purified from inflamed colons of WT and *Trpv1*^G564S+/+^ mice. (F) Relative mRNA expression of *Il1β*, *Il6*, *Il23*, and *Tnfα* in WT and *Trpv1*^G564S+/+^ DCs. (G) ELISA assay of IL-1β, IL-6, TNF-α, and IL-23 in supernatants of WT and *Trpv1*^G564S+/+^ DCs cultured *in vitro* for 24 h. (H–J) DCs were purified from spleens of WT and *Trpv1*^G564S+/+^ mice and stimulated with LPS (500 ng/mL) for 24 h. (H) Representative flow cytometry plots (top) and mean fluorescence intensity of CD80 and MHC II staining (bottom) in CD11c^+^ DCs. (I) ELISA assay of IL-1β, IL-6, TNF-α, and IL-23 in supernatants of WT and *Trpv1*^G564S+/+^ DCs. (J) Relative mRNA expression of *Il1β*, *Il6*, *Tnfα*, and *Il23* in WT and *Trpv1*^G564S+/+^ DCs. Data are representative of three independent experiments (mean ± SEM of four mice per group for one experiment). ∗p < 0.05, ∗∗p < 0.01, ∗∗∗p < 0.001.

We subsequently investigated which cell type within the hematopoietic pool was the main contributor to the enhanced susceptibility of *Trpv1*^G564S+/+^ mice to colitis. We focused on mononuclear phagocytes, including DCs and macrophages, because they are key components in anti-bacterial responses and presentation of antigen to T cells. First, we confirmed TRPV1 expression in CD11c^+^ DCs and CD11b^+^F4/80^+^ macrophages (Figure 2A). Consistent with our previous reports,^22^ no difference in TRPV1 expression was found in the cells from *Trpv1*^G564S+/+^ and WT naive mice, indicating that TRPV1 activity, but not protein level, was affected in *Trpv1*^G564S+/+^ cells. In the healthy state, we observed a comparable abundance of DCs and macrophages in colonic lamina propria (colonic-LP) of *Trpv1*^G564S+/+^ and WT mice (Figure 2B). DSS challenge induced the accumulation of DCs and macrophages in colonic-LP, with *Trpv1*^G564S+/+^ mice exhibiting a higher frequency of DCs as compared with WT littermates, whereas the frequency of macrophages has no significant difference between both groups (Figure 2B). Co-stimulatory molecule CD80 and MHC II are crucially involved in DC activation and antigen presentation during inflammation. There were increased frequencies of CD11c^+^CD80^+^ and CD11c^+^MHCII^+^ DC subsets in the inflamed colon of *Trpv1*^G564S+/+^ mice compared with WT littermates (Figures 2C and 2D). CD103^+^CD11b^+^ DCs are a subset unique to the intestine and the draining lymphoid nodes, which are most efficient at driving interleukin-6 (IL-6) production and mucosal Th17 cell responses.^23^ We found that the proportion of CD103^+^CD11b^+^ DCs was dramatically increased in the inflamed colon of *Trpv1*^G564S+/+^ mice (Figure 2E). To further determine the functional difference of DCs in *Trpv1*^G564S+/+^ and WT mice during colitis, we analyzed their cytokine profiles. Colonic-LP DCs sorted from *Trpv1*^G564S+/+^ mice expressed and secreted a higher level of IL-6, IL-1β, TNF-α, and IL-23 than did DCs from WT littermates (Figures 2F and 2G). Thus, TRPV1 gain of function alters the DC phenotype, inducing a more pro-inflammatory state and promoting colitogenesis.

We further purified DCs from spleens of mice and stimulated cells with lipopolysaccharide (LPS). As shown in Figure 2H, LPS led to higher expression of CD80 and MHC II on *Trpv1*^G564S+/+^ DCs compared with WT DCs. There was no significant difference of IL-1β and TNF-α expression and secretion between WT and *Trpv1*^G564S+/+^ DCs in response to LPS, whereas *Trpv1*^G564S+/+^ DCs expressed and secreted a significantly higher level of IL-6 and IL-23 than WT DCs (Figures 2I and 2J). These results indicated that TRPV1 hyperactivation in DCs renders them more sensitive to LPS-induced inflammatory responses.

### TRPV1 Gain of Function Influences Colonic CD4^+^ T Cell Homeostasis in DSS-Induced Colitis

**Figure 3:**
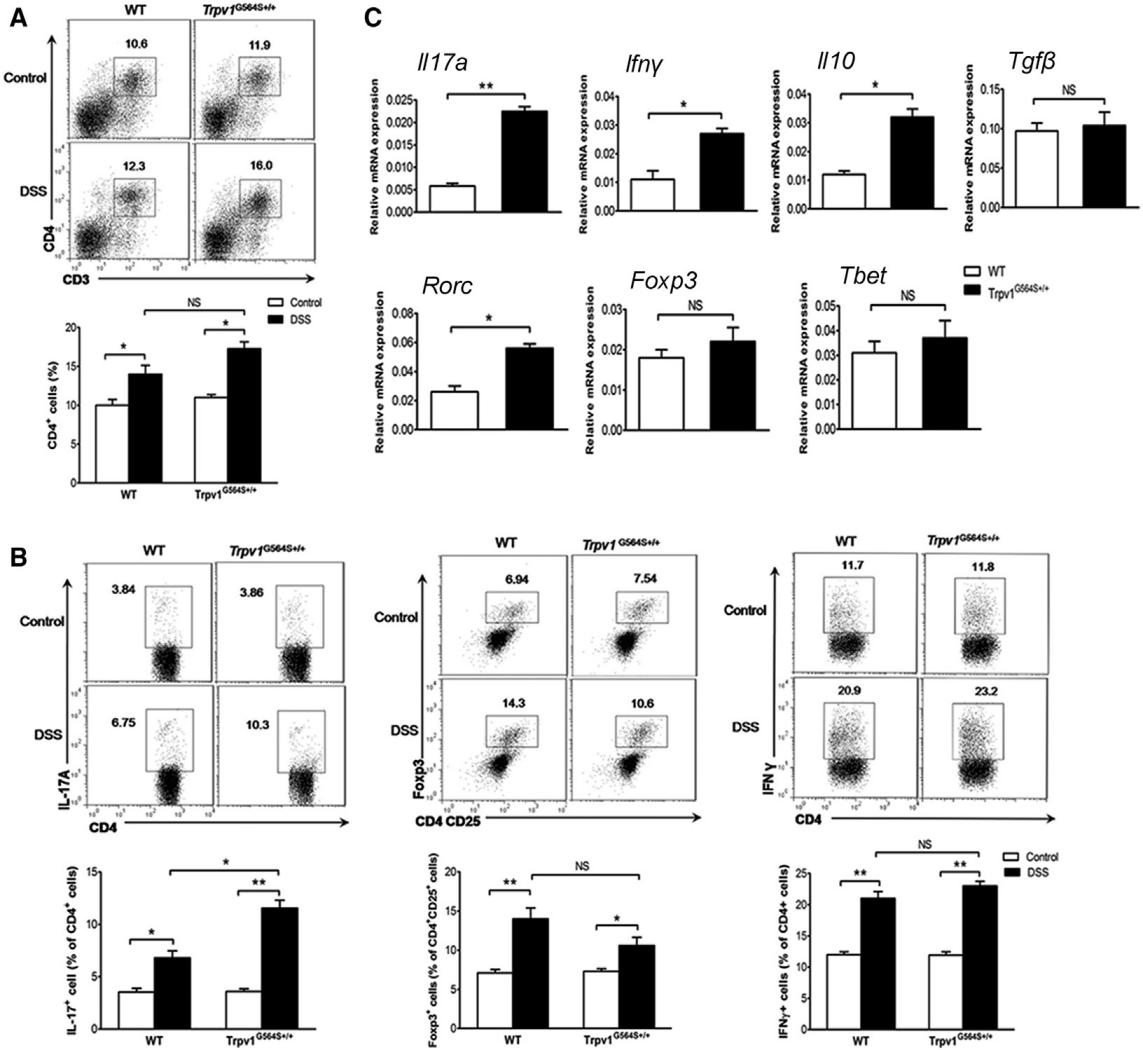
TRPV1 Gain of Function Influences Colonic CD4^+^ T Cell Homeostasis in Colitis Colonic lamina propria mononuclear cells were isolated from WT and *Trpv1*^G564S+/+^ mice treated with or without 2% DSS for 7 days. (A) Representative flow cytometry plots (top) and quantification (bottom) of CD3^+^CD4^+^ cells in total viable cells. (B) Representative flow cytometry plots (top) and quantification (bottom) of CD4^+^IL-17A^+^ (left) and CD4^+^IFNγ^+^ (right) cells in CD4^+^ cells and Foxp3^+^ cells in CD4^+^CD25^+^ cells (middle). (C) Relative mRNA expression of cytokines (*Il17a*, *Ifnγ*, *Il10*, and *Tgfβ*) and regulators of Th1 (*T-bet*), Th17 (*Rorc*), and Treg (*Foxp3*) differentiation in isolated CD4^+^ T cells from inflamed colons of WT and *Trpv1*^G564S+/+^ mice. Data are representative of three independent experiments (mean ± SEM of four mice per group for one experiment). ∗p < 0.05, ∗∗p < 0.01.

In addition to innate immune cells, CD4^+^ T cells play a critical role during colitogenesis. In the healthy state, the proportion of colonic-LP CD4^+^ T cells did not differ between *Trpv1*^G564S+/+^ and WT mice (Figure 3A), and there were similar percentages of IL-17A^+^, Foxp3^+^, and IFNγ^+^ cells among colonic-LP CD4^+^ T cells in *Trpv1*^G564S+/+^ and WT naive mice (Figure 3B). DSS challenge resulted in a minor increase in total CD4^+^ T cells within colonic-LP; however, no difference was found between *Trpv1*^G564S+/+^ and WT groups (Figure 3A). Notably, there was a significant increase in CD4^+^IL-17A^+^ cells in the inflamed colon of *Trpv1*^G564S+/+^ mice compared with WT littermates (Figure 3B). A decreased trend of CD4^+^Foxp3^+^ cells was observed in *Trpv1*^G564S+/+^-inflamed colons, although this was not statistically significant (Figure 3B). Moreover, DSS treatment led to an increase in CD4^+^IFNγ^+^ cells within colonic-LP, and this effect was comparable between *Trpv1*^G564S+/+^ and WT groups (Figure 3B). We further isolated CD4^+^ T cells from colonic-LP of DSS-treated mice and analyzed their cytokines and regulators of Th1/Th17/regulatory T cell (Treg) differentiation. As shown in Figure 3C, expression of *Il17a*, *Ifnγ*, and *Il10* was significantly increased in CD4^+^ T cells from *Trpv1*^G564S+/+^ mice compared with those from WT littermates, whereas *Tgfβ* level was comparable between both groups. Moreover, *Rorc*, the key transcription factor orchestrating Th17 polarization, was markedly increased in CD4^+^ T cells from *Trpv1*^G564S+/+^ mice compared with WT littermates (Figure 3C). The expression of transcription factors regulating Th1 (*T-bet*) and Treg (*Foxp3*) differentiation in CD4^+^ T cells did not differ between both groups (Figure 3C). Together with the finding that *Il17a* and Th17-ploarizing cytokines (*Il1β, Il6*, and *Il23*) were significantly upregulated in the inflamed colon of *Trpv1*^G564S+/+^ mice (Figure 1E), our results suggested that TRPV1 gain of function influences colonic CD4^+^ T cell homeostasis and appears to promote Th17 differentiation and consequently exacerbate colitis.

### *Trpv1*^G564S+/+^ DCs Require Inflammatory Signals to Promote Th17 Cell Differentiation

**Figure 4:**
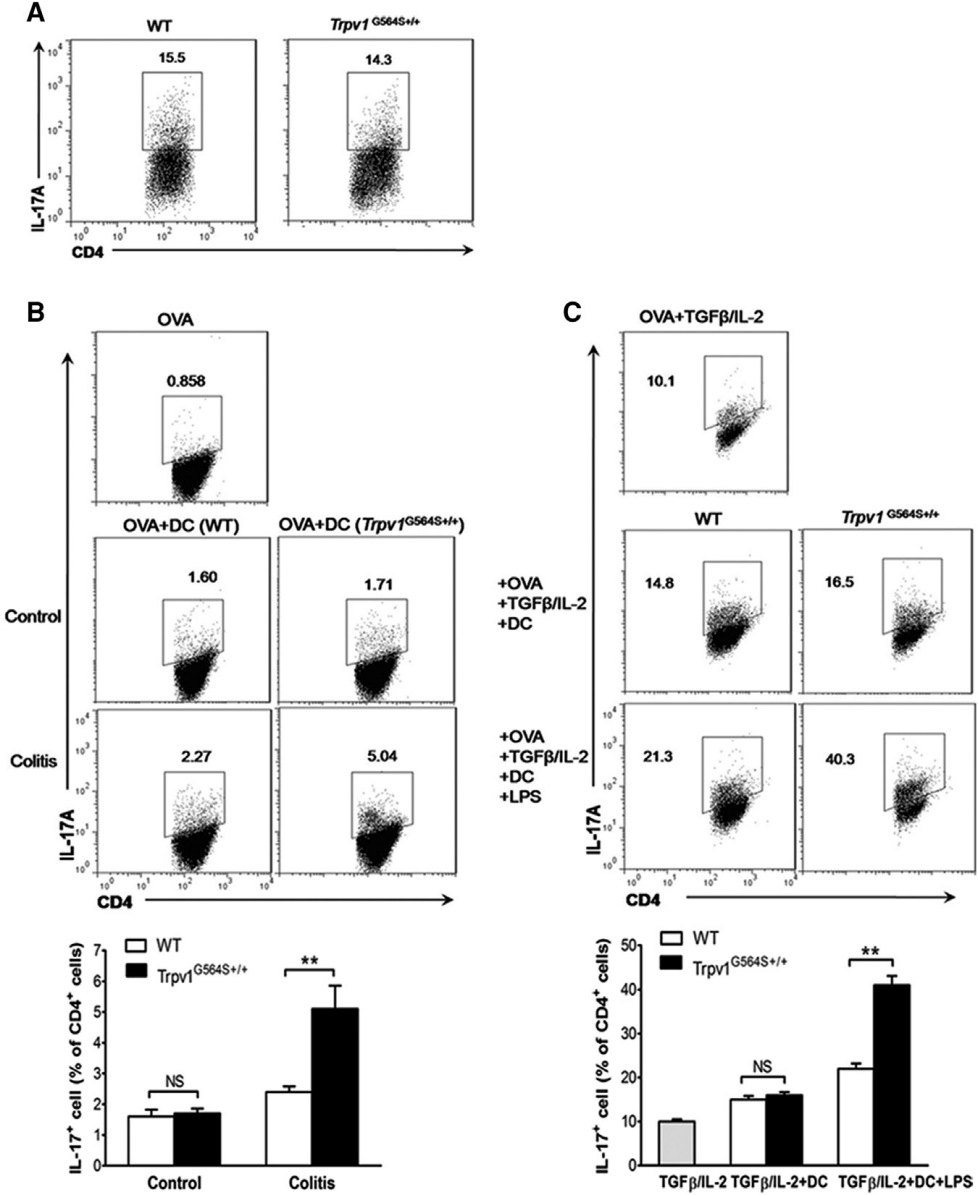
*Trpv1*^G564S+/+^ DCs Require Inflammatory Signals to Promote Th17 Cell Differentiation (A) Flow cytometry analysis of IL-17A expression in WT and *Trpv1*^G564S+/+^ naive T cells cultured *in vitro* for 5 days under Th17-polarizing conditions. (B) Naive OT-II T cells were cocultured with DCs from colons of WT or *Trpv1*^G564S+/+^ mice treated with or without DSS in the presence of OVA_323–339_ for 5 days. Representative flow cytometry plots (top) and frequency (bottom) of IL-17A^+^ population in CD4^+^ T cells. (C) Naive OT-II T cells were cocultured with LPS-stimulated WT or *Trpv1*^G564S+/+^ DCs in the presence of OVA_323–339_ and exogenous TGF-β and IL-2 for 5 days. Representative flow cytometry plots (top) and frequency (bottom) of IL-17A^+^ population in CD4^+^ T cells. Data are representative of three independent experiments (mean ± SEM of four mice per group for one experiment). ∗∗p < 0.01.

To identify the mechanism by which TRPV1 affected Th17 differentiation, we isolated naive CD4^+^ T cells from *Trpv1*^G564S+/+^ and WT mice and cultured them *in vitro* under Th17-polarizing conditions. As shown in Figure 4A, *Trpv1*^G564S+/+^ and WT naive CD4^+^ T cells differentiated similarly into Th17 cells, excluding the possibility of a T cell-intrinsic effect of TRPV1 hyperactivation on Th17 differentiation. Because DCs are the main APCs that trigger the activation and differentiation of T cells, we determined whether TRPV1 overactivation in DCs affected their capacity to induce Th17 differentiation. We used naive T cells from OT-II mice, which have transgenic expression of a T cell antigen receptor (TCR) specific for ovalbumin amino acids 323–339 (OVA_323–339_). DCs were sorted from the colonic-LP of *Trpv1*^G564S+/+^ and WT mice and co-cultured with naive OT-II cells. Colonic-LP DCs from *Trpv1*^G564S+/+^ and WT untreated mice induced limited Th17 cells *in vitro*, and there was no significant difference between both groups (Figure 4B). In contrast, DCs from the inflamed colon of *Trpv1*^G564S+/+^ mice induced more Th17 cells than did those from WT littermates (Figure 4B). Thus, *Trpv1*^G564S+/+^ DCs promoted Th17 differentiation under inflammatory conditions. This notion was further supported by culturing LPS-stimulated colonic-LP DCs together with naive OT-II cells. LPS enhanced the capacity of DCs to induce Th17 differentiation in the presence of transforming growth factor β (TGF-β) and IL-2 (Figure 4C). Of note, *Trpv1*^G564S+/+^ DCs stimulated with LPS induced a significantly higher frequency of Th17 cells than did those of WT DCs (Figure 4C). These were consistent with the finding that *Trpv1*^G564S+/+^ DCs secreted a higher level of Th17-ploarizng cytokines IL-6 and IL-23 than did WT DCs in response to LPS stimulation (Figure 2I). Together with the *in vivo* data, our results indicated that inflammatory signals are important in *Trpv1*^G564S+/+^ DCs to promote Th17 differentiation.

### TRPV1 Gain of Function Enhances Activation of Calcineurin/NFATc2 Signaling in DCs

**Figure 5:**
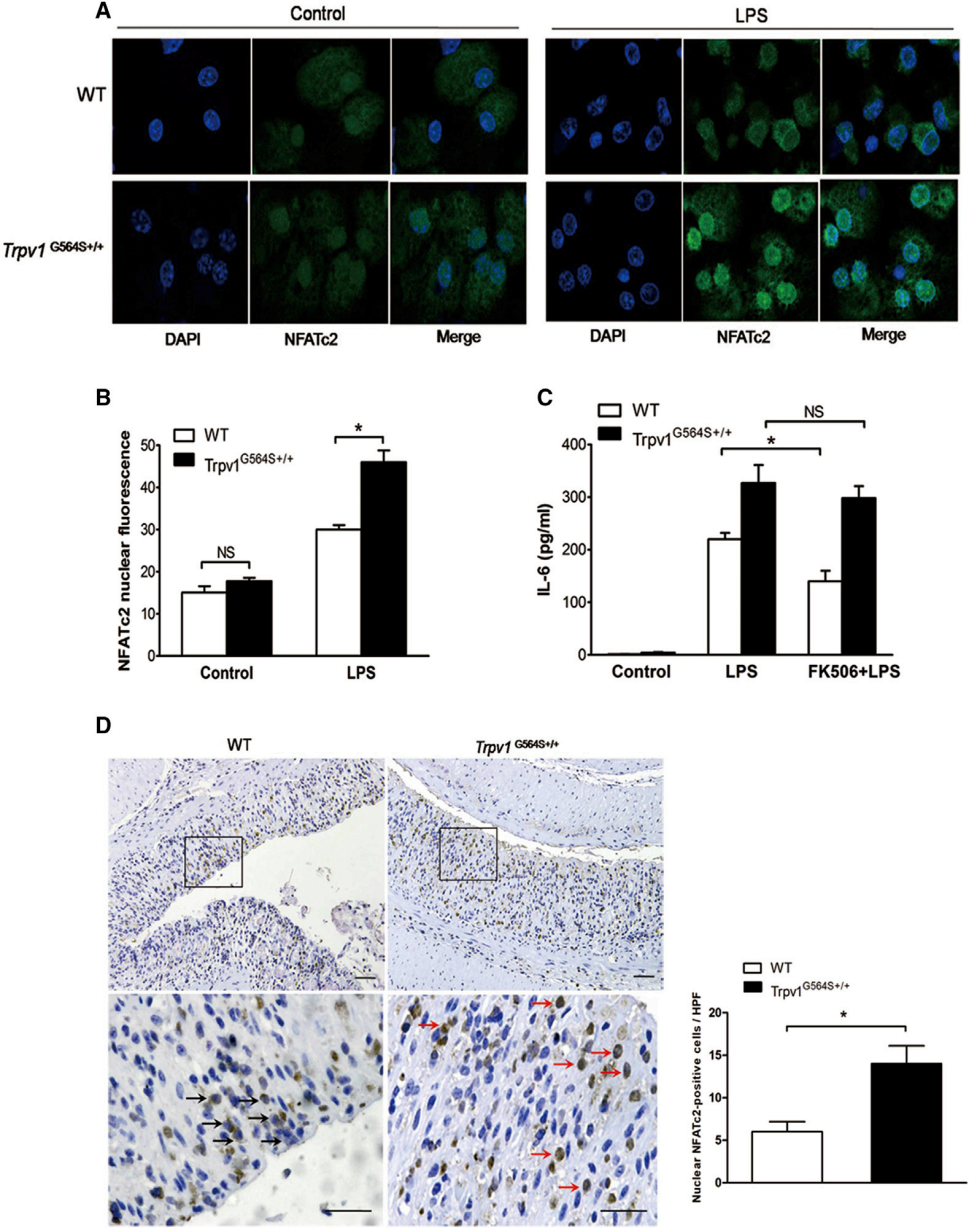
TRPV1 Gain of Function Enhances Activation of Calcineurin/NFATc2 Signaling in DCs (A) WT and *Trpv1*^G564S+/+^ BMDCs were left untreated or treated with LPS (1 μg/mL) for 1 h. Representative confocal immunofluorescence fields of NFATc2 staining (green). Cell nuclei were stained with DAPI. (B) Quantification of mean nuclear NFATc2 fluorescence of cells. Data represent mean ± SEM of at least 100 cells in three experiments. (C) ELISA assay of IL-6 in supernatants of WT and *Trpv1*^G564S+/+^ BMDCs stimulated with LPS (500 ng/mL) for 24 h in the presence or absence of FK506 (2 μmol/L). Data are representative of three independent experiments. ∗p < 0.05. (D) Immunohistochemical analysis of NFATc2 in colon sections of WT and *Trpv1*^G564S+/+^ mice treated with 2% DSS for 7 days (n = 6 per group). Black arrows indicate cells with cytosolic NFATc2 staining; red arrows indicate cells with nuclear NFATc2 staining. Quantification of nuclear NFATc2-positive cells is shown on the right. Data represent mean ± SEM, at least 50 high-power fields in each group. Scale bars, 50 μm.

TRPV1 activation controls intracellular Ca^2+^ level and triggers the calcineurin/NFAT signaling pathway, leading to cytokine secretion.^24^ We focused on NFATc2, the prominent isoform regulating DC activation.^25^ Because Toll-like receptor agonists can activate the calcineurin/NFATc2 cascade in DCs,^25^ we treated BM-derived DCs (BMDCs) with LPS and determined the kinetics of endogenous NFATc2 translocation. Immunofluorescence analysis showed a significant enhanced translocation of NFATc2 to the nucleus in *Trpv1*^G564S+/+^ BMDCs after LPS treatment (Figure 5A), and the mean nuclear NFATc2 fluorescence was markedly increased in *Trpv1*^G564S+/+^ BMDCs compared with WT BMDCs (Figure 5B). Moreover, the production of IL-6 was higher in *Trpv1*^G564S+/+^ BMDCs than WT BMDCs in response to LPS (Figure 5C). Treatment with the calcineurin inhibitor FK506 significantly inhibited LPS-induced IL-6 production in WT BMDCs, but not in *Trpv1*^G564S+/+^ BMDCs (Figure 5C). These data suggested that TRPV1 hyperactivation in DCs might promote calcineurin/NFATc2 signaling activation and enhance innate immune responses. We also investigated whether the calcineurin/NFATc2 cascade was altered in the colitis model. Immunohistochemical analysis showed much more localization of NFATc2 in the nucleus of infiltrating cells within inflamed colon of *Trpv1*^G564S+/+^ mice than WT littermates (Figure 5D). Thus, we speculated that TRPV1 gain of function exacerbates DSS-induced colitis, at least in part, through promoting NFATc2 activation in DCs.

### TRPV1 Is Highly Expressed in Patients with IBD

**Figure 6:**
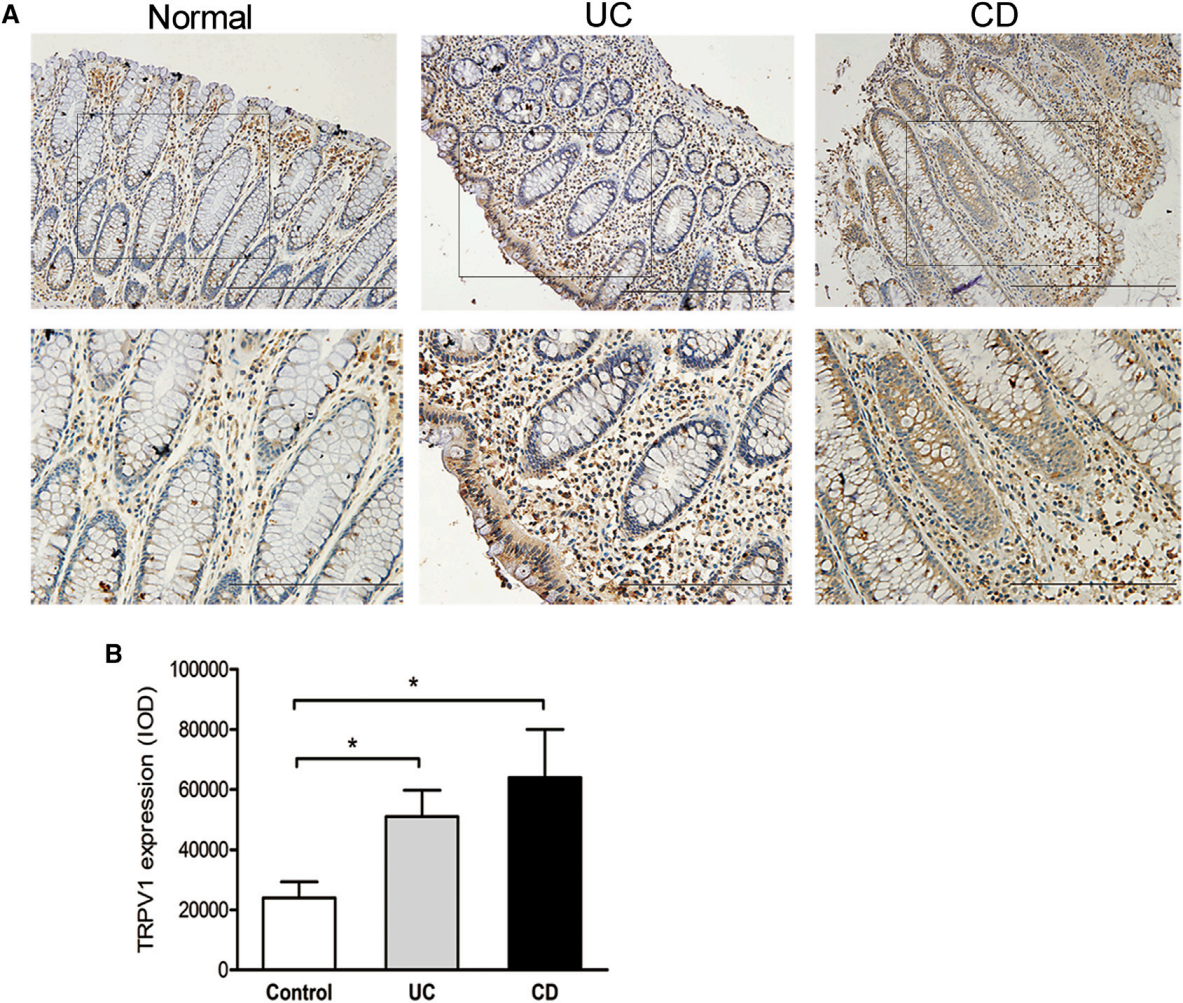
TRPV1 Is Highly Expressed in Patients with IBD (A) Representative immunohistochemical staining of TRPV1 in normal colon tissues (n = 30) and inflamed colon from UC (n = 34) and CD (n = 26) patients. Scale bars: 200 μm (top); 100 μm (bottom). (B) TRPV1 expression is quantified as integrated optical intensity (IOD). Data represent mean ± SEM. ∗p < 0.05.

Finally, we assessed whether our findings about TRPV1 in mice hold relevance for IBD patients. Previous studies on the expression of TRPV1 in inflamed human colon, particularly in non-neuronal tissues, have yielded contradictory results.^26^ Considering high expression of TRPV1 in nerve fibers, it is obvious that neurons largely contribute to the TRPV1 mRNA level detected in colon tissues. To examine the localization and expression of TRPV1 in non-neuronal components of colon, we performed immunohistochemical assay. In normal tissues, scattered expression of TRPV1 was observed in lamina propria cells, and weak expression was presented in epithelial cells (Figure 6A). Notably, TRPV1-positive cells were significantly increased in lamina propria of inflamed colon from UC and CD patients when compared with healthy controls (Figures 6A and 6B). TRPV1 expression in epithelial cells was remarkably enhanced in CD patients, but not in UC patients (Figure 6A). Because immune cells are major components in the lamina propria, it is tempting to speculate that a high level of TRPV1 in immune cells might be associated with IBD.

## Discussion

The role of ion channel TRPV1 in intestinal inflammation remains controversial. Here, by using a novel TRPV1 gain-of-function mouse model, we showed that TRPV1 hyperactivation significantly increased susceptibility to DSS-induced colitis. TRPV1 gain of function altered the number and phenotype of DCs in colon, resulting in elevated local pro-inflammatory cytokine levels and enhanced Th17 immune responses during colitogenesis. Mechanistically, TRPV1 gain of function in DCs increased activation of calcineurin/NFATc2 signaling in response to inflammatory stimuli. In patients with IBD, TRPV1 expression was significantly increased in lamina propria cells of inflamed colon compared with healthy controls. Our data identify a unique role for TRPV1 in regulating the activation and pro-inflammatory properties of DCs, highlighting the importance of TRPV1 in controlling mucosal immunity and IBD.

TRPV1 is highly expressed on sensory neurons throughout the gastrointestinal tract and is involved in abdominal pain associated with inflammation.^27^ TRPV1-positive nerve fibers are substantially increased in colon of IBD patients, which is positively correlated with abdominal pain severity.^9^^,^^10^ However, the impact of TRPV1 on IBD development is currently unknown. Discrepancies on the pro- or anti-inflammatory role of TRPV1 have been reported in animal studies. For example, both TRPV1 agonists and antagonists were shown to protect against experimental colitis.^20^^,^^21^^,^^28^ These opposing results may be caused by the different methods used to induce colitis and the non-TRPV1-specific effects of the agonists and antagonists. Conflicting results have also been reported in studies using a TRPV1-deficient mouse model for colitis.^17^, ^18^, ^19^ The TRPV1 overexpression model has been used but also has substantial limitations. Overexpression of TRPV1 in the absence of ligands might not be sufficient to display its function. To clarify the exact role of TRPV1 in intestinal inflammation, we exploited TRPV1 gain-of-function mutation mice and demonstrated that TRPV1 channel overactivation exacerbated DSS-induced colitis. It is interesting to note that *Trpv1*^G564S+/+^ mice did not develop spontaneous colitis, suggesting that endogenous TRPV1 channel activity is associated with susceptibility to inflammatory stimuli. Our concept that TRPV1 has a deleterious effect on colitogenesis is contrary to the results using TRPV1-deficient mice,^29^ implying that a homeostatic level of TRPV1 is important for mucosal protection, whereas overactivation of TRPV1 is proinflammatory.

TRPV1-expressing sensory neurons release neuropeptides, such as substance P (SP) and calcitonin gene-related peptide (CGRP), which participate in the neurogenic inflammation of gut. A recent study has shown that TRPV1 deficiency attenuated DSS-induced colitis with reduction in upregulation of SP-positive nerve fibers.^18^ In our study, there was no significant difference in the expression of SP and CGRP in the inflamed colon of *Trpv1*^G564S+/+^ and WT mice (data not shown), suggesting that neuropeptide-mediated mechanisms may not be critical to confer increased susceptibility of *Trpv1*^G564S+/+^ mice to colitis. TRPV1 is widely expressed in non-neuronal cells, including epithelial cells of gut and almost all types of immune cells.^6^^,^^13^^,^^14^^,^^21^ The wide distribution of TRPV1 suggests extended roles of TRPV1 in regulating inflammatory responses beyond neuropeptide release. Thus, we investigated the impact of TRPV1 gain of function on epithelial and immune cells during colitogenesis. Previous study has shown that TRPV4, another family member of TRP channels, is functionally expressed in intestinal epithelial cells, and its activation promotes cytokine release and colitis.^30^ Pharmacological activation of TRPV1 promotes apoptosis of epithelial cells.^20^ We therefore hypothesized that epithelial TRPV1 overactivation mediated the deleterious effects during colitis. However, by using BM chimeras, we found that TRPV1 gain of function in hematopoietic cells, but not in epithelial cells, was critical to exacerbate colon inflammation. This suggested that TRPV1 and TRPV4 might contribute to colitogenesis by different mechanisms. Moreover, the epithelial apoptosis is driven by the local inflammatory responses, rather than vice versa. Thus, colonic epithelial cells are not the primary targets for TRPV1 hyperactivation to confer enhanced susceptibility to colitis.

DCs act as an initiator and regulator of mucosal immunity.^2^ Accumulation of activated DCs has been observed in the inflamed sites of IBD patients, and DCs from IBD produce more pro-inflammatory cytokines than DCs from healthy controls.^3^^,^^31^ Animal studies also showed a detrimental role of DC aberrant activation in tissue injury and progression of colitis.^32^ TRPV1 is expressed on mouse and human DCs, but its function is controversial.^13^^,^^33^^,^^34^ In our study, *Trpv1*^G564S+/+^ mice exhibited significant accumulation of activated DCs in the inflamed colon. Moreover, *Trpv1*^G564S+/+^ DCs displayed upregulation of co-stimulatory molecules and cytokines in response to LPS stimulation *in vitro*. These results suggested that TRPV1 gain of function leads to DC aberrant activation and potentiates inflammatory responses in colitis.

DCs bridge innate and adaptive immunity, and therefore dysregulation of gut-infiltrated DCs could favor the development of effector T cells and result in unbalanced immune responses.^2^ Th17 cells have been implicated in the pathogenesis of human IBD and murine colitis.^35^ Cytokines produced by DCs, including IL-6, IL-1β, TGF-β, and IL-23, drive intestinal Th17 cell differentiation.^36^ In this study, *Trpv1*^G564S+/+^ mice exhibited significantly increased Th17 cells in the inflamed colon, which was associated with a higher level of IL-6, IL-1β, and IL-23. In addition to DCs, tissue-resident macrophages are also the main source of Th17-polarizing cytokines.^36^ We found similar amounts of macrophage in the inflamed site of *Trpv1*^G564S+/+^ and WT mice; thus, the increment of IL-6, IL-1β, and IL-23 in *Trpv1*^G564S+/+^ mice may be because of the accumulation and activation of DCs. Moreover, DCs isolated from the inflamed colon, but not normal colon, of *Trpv1*^G564S+/+^ mice were more potent to induce Th17 cell differentiation than DCs from WT controls, suggesting that inflammatory signals are critical to induce an efficient Th17-polarizing capacity in *Trpv1*^G564S+/+^ DCs. Published data have demonstrated an important role for TRPV1 in CD4^+^ T cell activation.^14^ Here, we found that Th17 differentiation *in vitro* was comparable in naive CD4^+^ T cells from *Trpv1*^G564S+/+^ and WT mice, indicating a selective role for TRPV1 in DC-mediated Th17 differentiation, but not in T cell-intrinsic Th17 differentiation. Because a systemic mutation of the TRPV1 model was used in this study, we could not exclude the possible roles of TRPV1 overactivation in other cell types affecting DC activation and Th17 responses during colitis. A model of a DC-specific mutation of TRPV1 would be helpful in our further studies.

Abnormal NFATc2 activation has been observed in colon of IBD patients.^37^ Moreover, NFATc2 deficiency suppresses CD4^+^ T cell-mediated colitis in mice.^38^ The calcineurin/NFATc2 pathway is remarkably active in innate immune cells after PRRs activation.^39^ Stimulation of DCs with LPS leads to Ca^2+^ influx and the subsequent calcineurin-dependent nuclear translocation of NFATc2.^25^^,^^39^ Here, we found much more nuclear localization of NFATc2 in colon-infiltrated cells of *Trpv1*^G564S+/+^ mice with colitis. Moreover, *Trpv1*^G564S+/+^ DCs showed enhanced NFATc2 nuclear translocation and subsequent IL-6 production in response to LPS stimulation. These data implied that sustained opening of TRPV1 channel impairs intracellular calcium homeostasis and calcineurin/NFATc2 cascade in DCs, and therefore increases the susceptibility to inflammatory stimuli. It is possible that TRPV1 gain of function could affect other signaling-mediated IL-6 production. However, we did not observe any difference in LPS-induced nuclear factor κB (NF-κB) and the kinases ERK1/2, JNK, and p38MAPK activation between *Trpv1*^G564S+/+^ and WT DCs (data not shown). Thus, TRPV1 regulates DC function, at least in part, through calcineurin/NFATc2 signaling.

The expression of TRPV1 in non-neuronal tissues of inflamed colon has been addressed in some studies with a small sample, but the results were contradictory.^26^ In our cohort, significantly enhanced TRPV1 protein expression has been shown in infiltrating inflammatory cells of active UC and CD patients compared with normal controls. Because TRPV1 can be activated or sensitized by inflammatory mediators and inflammation-associated tissue acidification, our finding suggested that TRPV1 might be a relevant target for IBD treatment. Owing to only expression changes detected in human samples, further studies are essential to investigate functional alterations of TRPV1 channel, as well as perform pharmacological interventions in IBD patients.

In summary, we identify an important role for ion channel TRPV1 in regulating mucosal DC activation and sustaining Th17 immune responses to inflammatory stimuli. TRPV1 might be a potential therapeutic target in controlling mucosal immunity and IBD.

## Materials and Methods

### Clinical Samples

Mucosal biopsy samples of inflamed colon were obtained from UC (n = 34) and CD (n = 26) patients in active phase of disease. Controls (n = 30) were selected from patients who were undergoing colonoscopy for other indications (such as cancer surveillance and polyp) and had a normal colon. Samples were fixed in 10% phosphate-buffered formalin until further analysis. All subjects received colonoscopies at Peking University First Hospital, and informed consents were obtained after the nature and possible consequences of the studies were explained. This study was carried out in accordance with the Declaration of Helsinki, and the protocol was approved by the Ethics Committee of Peking University First Hospital.

### Mice, Induction of Colitis, and BM Transplantation

The mice were kept under specific pathogen-free conditions at the Animal Center of Peking University First Hospital, and the animals’ care was in accordance with institutional guidelines. The mice were randomly grouped to minimize the subjective bias. As previously described,^22^ we used homologous recombination to generate mice with the specific site mutation c.G1690A in *Trpv1* on the C57BL/6 background, and this mutation led to a homozygous glycine-to-serine substitution at amino acid 564 (*Trpv1*^G564S+/+^). Acute colitis in *Trpv1*^G564S+/+^ and WT mice (male, 8–10 weeks) was induced by 2% (w/v) DSS (MP Biomedicals) in drinking water up to 7 days. The body weight of mice and clinical signs of colitis (diarrhea and rectal bleeding) were monitored daily. For BM transplantation, mice were subjected to 900 rad lethal total body irradiation and then intravenously reconstituted with 10^7^ BM cells prepared from the femurs and tibias of *Trpv1*^G564S+/+^ or WT mice. The chimeras were allowed to recover for 8 weeks, and then colitis was induced with 2% DSS. Mice from different genotypes were cage mixed during each experiment to minimize the influence of gut microbiota. OT-II TCR-transgenic mice recognizing chicken OVA_323–339_ in the context of I-Ab were bred in the Peking University First Hospital Animal Facility.

### Histopathological and Immunohistochemical Analyses

Paraffin-embedded colon sections from patients were subjected to immunohistochemical staining using anti-TRPV1 (Santa Cruz) at a dilution of 1:50. Colon sections from mice were stained with hematoxylin and eosin (H&E). Colitis activity score was determined based on previously published criteria^28^ by a pathologist blinded to the experimental groups. In brief, the total score ranged from 0 to 14, which represents the sum of scores from 0 to 4 for inflammation severity (0, none; 1, mild; 2, moderate; 3, severe), inflammation extent (0, none; 1, mucosa; 2, submucosa; 3, transmural), crypt damage (0, none; 1, basal 1/3; 2, basal 2/3; 3, crypt lost, surface epithelium present; 4, crypt and surface epithelium lost), and percent involvement (0, 0%; 1, 1%–25%; 2, 26%–50%; 3, 51%–75%; 4, 76%–100%). NFATc2 immunohistochemical analysis was performed by using anti-NFATc2 (Santa Cruz) at a dilution of 1:100. Integrated optical density (IOD) values were measured by Image-Pro Plus 6.0 image processing software (Media Cybernetics).

### Quantitative RT-PCR (qRT-PCR)

Total RNA was extracted from mouse colon tissues and cells using the Oligotex mRNA Mini Kit (QIAGEN) and TRIzol reagent (Invitrogen), respectively. cDNA was synthesized using the High Capacity cDNA Reverse Transcription Kits, and qRT-PCR was performed using Power SYBR Green PCR Master Mix (Applied Biosystems). Gene expression was calculated relative to that of *Gapdh*. The sequences of primers are as follows: *Foxp3* forward 5′-CACCTATGCCACCCTTATCCG-3′ and reverse 5′-CATGCGAGTAAACCAATGGTAGA-3′; *Gapdh* forward 5′-AGGTCGGTGTGAACGGATTTG-3′ and reverse 5′-TGTAGACCATGTAGTTGAGGTCA-3′; *Ifnγ* forward 5′-ATGAACGCTACACACTGCATC-3′ and reverse 5′-CCATCCTTTTGCCAGTTCCTC-3′; *Il1β* forward 5′-GCAACTGTTCCTGAACTCAACT-3′ and reverse 5′-ATCTTTTGGGGTCCGTCAACT-3′; *Il6* forward 5′-TAGTCCTTCCTACCCCAATTTCC-3′ and reverse 5′-TTGGTCCTTAGCCACTCCTTC-3′; *Il10* forward 5′-GCTCTTACTGACTGGCATGAG-3′ and reverse 5′-CGCAGCTCTAGGAGCATGTG-3′; *Il12* forward 5′-AGACATCACACGGGACCAAAC-3′ and reverse 5′-CCAGGCAACTCTCGTTCTTGT; *Il17a* forward 5′-TTTAACTCCCTTGGCGCAAAA-3′ and reverse 5′-CTTTCCCTCCGCATTGACAC-3′; *Il23a* forward 5′-CAGCAGCTCTCTCGGAATCTC-3′ and reverse 5′-TGGATACGGGGCACATTATTTTT-3′; *Rorc* forward 5′-GACCCACACCTCACAAATTGA-3′ and reverse 5′-AGTAGGCCACATTACACTGCT-3′; *T-bet* forward 5′-AGCAAGGACGGCGAATGTT-3′ and reverse 5′-GGGTGGACATATAAGCGGTTC-3′; *Tgfβ* forward 5′-CCACCTGCAAGACCATCGA-3′ and reverse 5′-CTGGCGAGCCTTAGTTTGGAC-3′; *Tnfα* forward 5′-CCCTCACACTCAGATCATCTTCT-3′ and reverse 5′-GCTACGACGTGGGCTACAG-3′.

### Cell Isolation and Flow Cytometry Analysis

The mouse spleens were removed and crushed through a 40-μm cell strainer, and red blood cells were lysed using ammonium-chloride-potassium (ACK) Lysing Buffer (Invitrogen). Lamina propria mononuclear cells were isolated from colonic tissues as described previously.^40^ In brief, the epithelial layer of colon was removed using two rounds (20 min each) of EDTA (5 mmol/L) and DTT (1 mmol/L) rotating at 37°C. After washing, the remaining tissues were cut into ∼1-mm pieces and digested with collagenase IV (0.5 mg/mL; Sigma-Aldrich), DNase I (0.5 mg/mL; Sigma-Aldrich), and Dispase II (3 mg/mL; Roche) for 20 min rotating at 37°C. This process was repeated to ensure all visible pieces were fully digested. The cell mixture was filtered through a 70-μm strainer, washed with PBS, and the immunocytes were separated by Percoll density gradient centrifugation. For cell surface antigens staining, single-cell suspensions were stained with fluorochrome-conjugated antibodies for 30 min at 4°C. For intracellular cytokine staining, cells were incubated with the phorbol 12-myristate 13-acetate (PMA)/ionomycin (Sigma-Aldrich) plus brefeldin A (eBioscience) for 4 h, and then fixed and permeabilized for 20 min at 4°C (Intracellular Fixation & Permeabilization Buffer; eBioscience). Intracellular Foxp3 staining was performed using Foxp3/Transcription Factor Staining Buffer Set (eBioscience). The following monoclonal anti-mouse antibodies were used: anti-CD3-PE (phycoerythrin), anti-CD4-allophycocyanin, anti-CD4-PE-Cy7, anti-CD11b-fluorescein isothiocyanate, anti-CD11b-PE-Cy7, anti-CD11c-allophycocyanin, anti-CD11c-Alexa Fluor 700, anti-MHC II-fluorescein isothiocyanate (FITC), anti-F4/80-PE, anti-CD103-PerCP-eFlour 710, anti-CD80-PE, anti-CD25-PE, anti-Foxp3-PeCy5.5, anti-IFNγ-PE, and anti-IL-17A-PE (all from eBioscience). Isotype-matched antibodies were used as controls. For TRPV1 staining, cells were fixed with 2% paraformaldehyde for 10 min at 37°C and permeabilized in chilled methanol for 30 min at 4°C. The cells were incubated with anti-TRPV1 (Alomone Labs) or isotype rabbit IgG as control for 1 h at room temperature and then stained with Alexa Fluor 488-conjugated goat anti-rabbit IgG (Abcam). Samples were analyzed on a BD Influx (BD Biosciences), and the data were analyzed using FlowJo software (Tree Star).

### Cell Purification

DCs and total CD4^+^ T cells were isolated from spleens or colonic-LP of mice using the Pan Dendritic Cell Isolation Kit and CD4^+^ T Cells Isolation Kit (all from Miltenyi Biotec), respectively. Naive CD4^+^ T cells were isolated from spleens of mice using a Naive CD4^+^ T cell Isolation Kit (Miltenyi Biotec). Cell purity was assessed by flow cytometry and was consistently above 95%.

### BMDCs and Immunocytochemical Analysis

BM cells were obtained from femurs and tibias of *Trpv1*^G564S+/+^ or WT mice and cultured in RPMI 1640 medium supplemented with 10% FBS, 1% penicillin-streptomycin, 2 mM L-glutamine, 50 mM 2-mercaptoethanol (all from Invitrogen), and 20 ng/mL recombinant mouse granulocyte-macrophage colony stimulating factor (GM-CSF) (PeproTech). On day 10, nonadherent cells were collected, and CD11c^+^ BMDCs were isolated using mouse CD11c microbeads (Miltenyi Biotec). For immunocytochemical assay, BMDCs were fixed in 2% paraformaldehyde and spun onto slides. Cells were permeabilized in chilled methanol and then blocked with anti-CD16/CD32 (BD Pharmingen). Anti-NFATc2 (Santa Cruz) was applied overnight at 4°C followed by incubation with Alexa Fluor 488-labeled secondary antibody (Abcam). Nuclei were visualized with DAPI staining.

### *In Vitro* Th17 Differentiation

Naive CD4^+^ T cells from *Trpv1*^G564S+/+^ or WT mice were cultured in a 48-well plate coated with anti-CD3 (5 μg/mL) and anti-CD28 (1 μg/mL) antibody in the presence of IL-6 (20 ng/mL), IL-23 (10 ng/mL), and TGF-β (2 ng/mL). In co-culture experiments, naive CD4^+^ T cells from OT-II mice were stimulated with OVA_323–339_ (2.5 μg/mL) and mixed with lamina propria DCs from *Trpv1*^G564S+/+^ or WT mice either stimulated with or without TGF-β (2 ng/mL) + IL-2 (5 ng/mL) or LPS (1 μg/mL) + TGF-β (2 ng/mL) + IL-2 (5 ng/mL) (all cytokines from PeproTech). After 5 days of culture, cells were harvested and restimulated with PMA/ionomycin plus brefeldin A for intracellular IL-17A staining.

### Cytokine Production and Enzyme-Linked Immunosorbent Assay (ELISA)

Cytokines IL-6, IL-1β, TNF-α, and IL-23 in the cell culture supernatant were quantified with ELISA kits following manufacturer’s instructions (eBioscience).

### Statistical Analysis

Data are expressed as mean ± SEM. All statistical analyses were performed using GraphPad Prism 5 software. Difference was analyzed by parametric (Student’s t test) or nonparametric (Mann-Whitney *U* or Wilcoxon test) test. A p value <0.05 was considered statistically significant.

## Author Contributions

Y.D., Y.Y., and W.W. conceived and designed the experiments. L.D., T.W., Z.K., L.H., and C.W. performed the experiments and/or analyzed data. G.T. and W.Z. collected clinical samples. Q.G. provided technical and material support and helped with the experiments. L.D. and Y.D. wrote and edited the paper. Y.D. and Y.Y. provided funding and overall project supervision.

## Conflicts of Interest

The authors declare no competing interests.
